# Dynamic Cellular Regulation of Proteasome Translocation and Phase Transition

**DOI:** 10.3390/biology15151253

**Published:** 2026-07-30

**Authors:** Conner Butcher, Jianhui Li

**Affiliations:** Department of Biomedical Engineering and Science, Florida Institute of Technology, Melbourne, FL 32901, USA; cbutcher2019@my.fit.edu

**Keywords:** ubiquitin–proteasome system, nucleocytoplasmic translocation, proteasome translocation dynamics, liquid–liquid phase separation, proteasome condensates, autophagy, proteostasis, metabolic stress

## Abstract

Proteasome dysfunction has been implicated in the pathogenesis of many human diseases, and the proteasome system has emerged as a major therapeutic target for cancer treatment. Besides proteasome activity, the dynamics of proteasome localization provide another tier of mechanism for regulating proteasome function and cellular protein homeostasis. While proteasomes are highly enriched in the nucleus, they can dynamically reshuffle across cellular compartments in response to metabolic cues. This localization shift is coupled with autophagy as a major mechanism for cell survival under stress. Under certain metabolic stress, proteasomes can reorganize into distinct membraneless condensates, termed proteasome condensates. While the current understanding of the biological significance of proteasome condensates is limited, they may provide proteolytic control to meet metabolic needs under stress. This review highlights how distinct metabolic cues, such as carbon starvation, amino acid deficiency, and senescence, regulate divergent proteasome fates including proteasome subcellular translocation, autophagic degradation of proteasomes, and proteasome condensate formation. A better understanding of proteasome regulation in a spatiotemporal manner will help identify new therapeutic targets for diseases affected by proteasome dysfunction.

## Simple Summary

The proteasome is a large macromolecular complex that manages cellular protein degradation. While proteasomes are normally located in the information center of the cell, the nucleus, they can move to other compartments depending on the metabolic needs of the cell. During carbon starvation, proteasomes can move from the nucleus to the cytoplasm, where they form densely packed membraneless condensates, termed proteasome condensates. *In situ* atomic-level tomography of proteasome condensates has shown that proteasome monomers can form trimers as packaging blocks to assemble paracrystalline arrays in proteasome condensates. Despite recent structural advancement, there are still gaps in understanding how and why proteasomes move among different cellular compartments and why they reorganize into proteasome condensates under certain metabolic conditions. This review explores the causes and effects of proteasome movement in the cell during environmental disruptions. Understanding the underlying mechanisms of proteasome translocation may provide insights into improved therapies for diseases related to proteasome function.

## 1. Introduction

Protein homeostasis, also known as proteostasis, is a tightly regulated biological process that maintains the balance of cellular proteins and prevents the accumulation of misfolded or damaged proteins in cells [1]. Accumulation of misfolded or damaged proteins has been implicated in the pathogenesis of many hallmark diseases, such as Alzheimer’s and Parkinson’s diseases [2,3]. Therefore, the cell has developed multilayered protein quality-control systems to prevent the accumulation of aberrant proteins throughout the life cycle of proteins, from biogenesis to degradation. Molecular chaperones, including heat shock proteins (Hsps), regulate protein folding by correcting misfolded proteins and assisting proper folding during protein biogenesis [4]. Meanwhile, the cell deploys anti-apoptotic pathways that degrade aberrant proteins or large protein aggregates and complexes to maintain proteostasis and cell survival under stress [5]. Specifically, cells utilize two major degradation systems to control protein turnover and provide cellular protection against aberrant protein accumulation: autophagy and the ubiquitin–proteasome system (UPS) [6].

Rather than operating independently, autophagy and the UPS function coordinately in response to metabolic stressors. While the UPS typically handles the majority of protein turnover [7], autophagy acts as a complementary system for large-scale clearance of protein complexes and organelles [8]. Autophagy is classified into three distinct types: macroautophagy, microautophagy, and chaperone-mediated autophagy [9]. Moreover, autophagy can also be categorized into two additional features for substrate recognition: nonselective and selective autophagy [10,11]. Nonselective autophagy focuses on the bulk breakdown of cytoplasmic components in response to metabolic shifts [12]. Selective autophagy requires cargo receptors that mediate the specificity of targeted cellular cargo [13]. Macroautophagy involves the autophagosome, a double-membraned vesicle, to shuttle cargos in bulk to the lysosome in mammalian cells or the vacuole in yeast and plant cells, where the two structures fuse to create an autolysosome for hydrolytic breakdown of cargo components [14]. Microautophagy is similar in function to macroautophagy, but instead of utilizing intermediary autophagosomes, the limiting lysosome or vacuole membrane directly engulfs the substrate cargos via invagination [15]. Both macroautophagy and microautophagy can be selective or nonselective depending on metabolic cues. Chaperone-mediated autophagy (CMA) is a selective autophagy pathway that forgoes the use of vesicles and instead translocates the targeted cytosolic proteins directly to the lysosome for degradation [16].

Working concurrently with autophagy, the UPS is a conserved multi-component system responsible for the removal of targeted cellular proteins [7]. Ubiquitin tags protein substrates through ubiquitylation, which acts as a canonical signal that drives UPS-mediated protein degradation. The ubiquitylation cascade requires three classes of enzymes: ubiquitin-activating enzyme (E1), ubiquitin-conjugating enzyme (E2), and ubiquitin ligase (E3). Ubiquitin activation is initiated by E1, which adenylates ubiquitin using ATP, forming a thioester bond between ubiquitin and the E1 active-site cysteine. Ubiquitin is transported to the substrate via E2. Upon arrival, E3 will act as a transient docking platform to attach the ubiquitin to the substrate [17]. Ubiquitin can form diverse configurations, such as mono-ubiquitylation and K48- and K63-linked polyubiquitylation. Ubiquitin chain conformation and length play an important role in substrate recognition by the proteasome [18,19]. After specific ubiquitylation of protein substrates, they are ultimately recognized and degraded by the proteasome [20].

The proteasome is an evolutionarily conserved, ~2.5 MDa multisubunit complex responsible for the recognition and degradation of ubiquitylated substrates. The 26S proteasome holoenzyme is composed of two major components: the 19S regulatory particle (RP) and the 20S core particle (CP) [21,22]. The RP is divided into two subcomplexes, the lid and base. The lid comprises Rpn3/5-7/9/12 (proteasome COP9 signalosome initiation factor 3 (PCI) domain proteins for structural support), Rpn8 (non-ATPase regulatory component), Rpn11 (deubiquitinase), and Sem1. The base comprises a heterohexameric ring of six AAA+ ATPases (Rpt1-6) that anchors the ubiquitin receptors Rpn10 and Rpn13, along with Rpn1 and Rpn2, which coordinate the shuttle factors Rad23 and Dsk2 [20,23]. During substrate engagement, the base ATPases utilize ATP-dependent hydrolysis to unfold and mechanically translocate substrates into the CP central chamber for degradation [24]. The CP is responsible for the catalytic activity of the proteasome, composed of two outer heptameric α rings (α1–7) and two inner heptameric β rings (β1–7). While the α rings function as a structural gatekeeper for the CP, the β rings are responsible for the proteolysis of substrate proteins [25]. β rings harbor three types of proteolytic activity: caspase-like activity (β1) cleaves peptide bonds after acidic residues (e.g., aspartate or glutamate), trypsin-like activity (β2) cleaves peptide bonds after basic residues (e.g., lysine or arginine), and chymotrypsin-like activity (β5) cleaves peptide bonds after bulky hydrophobic residues (e.g., tyrosine, phenylalanine, tryptophan, leucine, or isoleucine). While each proteolytic activity is sufficient on its own, the three working in tandem provide greater efficiency [25,26].

## 2. Proteasome Localization Dynamics

Proteasomes are highly dynamic complexes that can redistribute intracellularly based on metabolic needs [27]. For efficient cellular gatekeeping, cellular compartments are separated by intracellular membranes. The nuclear envelope (NE) serves as a gatekeeper, ensuring the nucleus maintains faithful regulation of genetic material. The NE is composed of the inner and outer nuclear membranes. These membranes are made of phospholipid bilayers that separate the cytoplasmic contents from the nucleus [28]. The two membranes are connected by the nuclear pore complex (NPC), a massive multisubunit complex (∼110 MDa) comprising multiple copies of ∼34 distinct proteins. The NPC acts as a bidirectional molecular channel to filter through small polar molecules and macromolecules [29,30]. While the definitive structure of the NPC slightly varies among species, the overall structural design remains consistent. The NPC comprises two classes of nucleoporin proteins (Nups): the asymmetric and symmetric Nups [31]. The asymmetric Nups are nucleoporins that attach to the cytoplasmic and nuclear faces of the core NPC. These structures are known as cytoplasmic filaments and the nuclear basket. The symmetrical Nups consist of an outer ring coat, an inner ring coat, an inner ring, and pore membrane proteins (POMs) [29]. At the core of the NPC lies an array of Nups with phenylalanine–glycine (FG) repeat domains that function as a permeability barrier, facilitating the bidirectional diffusion of molecules [32]. The cargoes can be transported through the NPC by passive and facilitated diffusion. Small molecules up to ∼5 nm can diffuse freely through the NPC via passive diffusion. Facilitated diffusion is initiated by cargo proteins interacting with nuclear transport receptors (NTRs), specifically karyopherins (importins and exportins), which bind FG-Nups to initiate transport. The NTRs recognize nuclear localization signals (NLSs) or nuclear export signals (NESs) that are displayed by the cargos to initiate transport [33].

Proteasome localization dynamics are relatively conserved in eukaryotic cells, with proteasomes translocating among subcellular compartments to meet metabolic needs. Although translocation tendencies can vary with cell types and metabolic cues, proteasomes are highly enriched in the nucleus in yeast, plant, and human cells [34,35]. During shifts in metabolic aptitude, proteasomes can relocate and reorganize within the cell depending on the type of stressor. Under carbon starvation conditions, cells will enter a non-proliferating state known as quiescence, which is directly related to changes in proliferation and metabolic state. This metabolic shift can result in the nucleocytoplasmic translocation of proteasomes and, in some instances, the formation of membraneless condensates [36]. Proteasome translocation is highly regulated by multiple factors, including cell model type, energy status (ATP/pH), ubiquitylation, mTOR signaling, and liquid–liquid phase separation (LLPS) drivers [37,38,39,40,41]. Proteasome translocation can be classified into three categories: nuclear, cytoplasmic, and vacuolar. The intricacy of proteasome translocation dynamics remains underinvestigated. For example, under carbon starvation conditions, proteasomes can be translocated from the nucleus to the cytoplasm and reorganized into condensates and sorted to the vacuole for autophagic degradation in yeast cells [37]. However, the cellular factors that mediate proteasome nucleocytoplasmic translocation and determine their final destination remain unclear, as do the factors that facilitate their shuttling across the nuclear membrane.

### 2.1. Proteasome Nucleocytoplasmic Translocation

Under homeostatic conditions, proteasomes are primarily localized in the nucleus, contributing to the overall maintenance of nuclear functions, such as transcription control, DNA damage repair, cell cycle progression, and protein quality control (PQC) [42,43,44,45]. In yeast, the UPS is a key contributor to inner nuclear membrane-associated degradation and San1-mediated PQC, which maintain proteostasis in the nucleus [46]. Despite the nuclear localization of most proteasomes, CP biogenesis begins in the cytoplasm, where the 7 different α ring subunits are arranged into a heptameric ring with assistance from two heterodimeric chaperone proteins, Pba1–2 and Pba3–4. Once the α ring is completed, the 7 different β subunits assemble on the inner surface of the α ring with the assistance of Ump1 (human proteasome maturation protein, POMP), creating a “half-proteasome” with a single α and β ring [22]. The “half-proteasomes” are then shuttled into the nucleus, where they dimerize into preholoproteasomes during CP maturation [47]. To shuttle from the cytoplasm to the nucleus, the proteasome requires Blm10 and the canonical Sts1/Srp1/Kap95 karyopherin-α/β pathway. Blm10 is also used in the import of mature proteasomes [48]. Blm10 is a large ~240 kDa protein that attaches directly to nascent α rings, acting as a gate guardian, preventing premature proteasome activity at the NPC. Additionally, Blm10 acts as an intermediary with the FG-Nups located at the NPC, shuttling proteasomes across the nuclear membrane [49]. The canonical Sts1/Srp1/Kap95 karyopherin-α/β pathway functions similarly to Blm10. Sts1’s C-terminus binds directly to the RP, while the N-terminus region, which contains the NLS, binds to Srp1 (karyopherin-α), which then recruits Kap95 (karyopherin-β) to move through the NPC to the nucleus [50,51].

In mammalian cells, AKIRIN2, a transcriptional regulator, plays an important role in the nuclear import of fully assembled 20S proteasomes. Functionally, AKIRIN2’s C-terminus binds directly to the α ring, occupying the binding sites for the gate-opening 19S-ATPases, preventing non-selective degradation during nuclear transport [52,53]. AKIRIN2 functions in a similar way to Sts1 to interact with importins for nuclear import of proteasomes [53]. Nuclear export of the proteasome may initiate proteasome recycling and redistribution. While the mechanisms of proteasome export are unclear, the Crm1 (exportin-1) and UBIN-POST systems are vital for the canonical nuclear export of ubiquitylated proteins to the cytosol for nuclear proteostasis in mammalian cells [54]. However, it remains unclear whether these export factors are involved in every event of proteasome nuclear export or are dedicated to canonical proteasome transport under homeostatic conditions. Further studies are needed to understand the key components involved in proteasome nuclear export.

Nuclear translocation of the proteasome under stress conditions promotes the advancement of proteostasis for cell survival. While proteasomal distribution under certain metabolic shifts in yeast leads to the movement of proteasomes from the nucleus to the cytoplasm, other examined stress conditions result in the reorganization of the majority of nuclear proteasomes. Cellular senescence, or organismal aging, is initiated by decreased cellular viability, accompanied by increased DNA damage rate, increased levels of reactive oxygen species (ROS), and morphed cellular structure [55]. In addition to decreased cell viability, proteasome function declines considerably, correlating with increased cellular vulnerability and accumulation of aberrant proteins [56]. In mice, removal of senescent cells via apoptosis prevented or delayed tissue dysfunction and extended lifespan [57]. Moreover, senescence stress induced the decreased accumulation of old proteasomes within the nucleus [58]. In human cells, proteasomes were associated with nuclear foci, known as senescence-associated nuclear proteasome foci (SANPs), during prolonged senescence [59]. However, further investigation will be required to confirm that shifts in metabolic aptitude are the root cause of proteasome relocation within the nucleus. Similar to senescence, prolonged amino acid deprivation induced the formation of proteasome-containing condensates, denoted as starvation-induced proteasome assemblies in the nucleus (SIPAN), near the nuclear periphery [40]. Still, further information is required to determine the exact motives underlying proteasome nuclear translocation dynamics and to identify the factors that contribute to differential localization dynamics across stress conditions, such as senescence and amino acid starvation.

Cytoplasmic translocation of the proteasome is an under-explored topic in proteasome trafficking research. The canonical export receptor Crm1/Xpo1 is implicated in the nuclear export of proteasomes across the NPC. Leptomycin B, a potent and specific nuclear export inhibitor, inhibits the Crm1/Xpo1 pathway and proteasome nuclear export in mammalian cells [60,61]. Whether the Crm1/Xpo1 export machinery is applicable to all events of cytoplasmic proteasome translocation remains to be elucidated. Additional studies will be required to paint a complete picture of Crm1/Xpo1’s role in proteasome cytoplasmic translocation and the functions this machinery mediates. In most instances, proteasome translocation to the cytoplasm precedes either proteasome condensate formation or movement into the vacuole for autophagic degradation. Under carbon starvation, nuclear proteasomes form trimers and higher-order structural arrangements for the assembly of proteasome condensates, namely proteasome storage granules (PSGs), in the cytoplasm [62,63]. During the cytoplasmic translocation of nuclear proteasomes, normal proteasomes congeal into reversible PSGs in yeast cells [64]. The exact mechanism by which proteasomes are shuttled via higher-order arrangements remains unclear. Proteasome lid components such as Sem1, an intrinsically disordered protein, play an important role in the nuclear export of proteasomes during carbon starvation, though more studies will be required to gain insights into proteasome self-regulation [37]. In a multitude of other stressors, proteasomes exit the nucleus into the cytoplasm for proteasomal recycling and degradation via autophagy. This transition can be found in stressors, such as senescence, amino acid deprivation, and carbon starvation [37,38,58].

### 2.2. Vacuolar Translocation of Proteasome by Autophagy

Proteasome efficiency is required for proteostasis. In response to nutrient starvation, proteasomes can be degraded and recycled for the upkeep of proteostasis [65]. During nitrogen starvation, proteasomes are shuttled from the nucleus to the cytoplasm for vacuolar degradation via Atg8-mediated autophagy [66]. Interestingly, nuclear proteasomes partially disassemble prior to transport for autophagic turnover, while prior cytosolic proteasomes remain untouched [67]. Target of rapamycin complex 1 (TORC1) is a master nutrient sensor involved in the regulation of proteasome levels. When TORC1 was inhibited by the chemical inhibitor rapamycin, proteasome levels increased following a decrease mediated by proteasome autophagy. However, despite nitrogen starvation also inhibiting TORC1, the rate of proteasome autophagy was increased without the initial upregulation of proteasome levels [68]. This evidence suggests that there are additional cellular factors required for efficient proteasome turnover beyond general autophagy, including the selective autophagy receptor Atg11 and the MAP kinase pathway [68].

While the distinctions among pathways are still being researched, there are noticeable differences among distantly related species. In the model plant *Arabidopsis thaliana* (*A. thaliana*), the proteasome RP subunit Rpn10 interacts with both Atg8 and ubiquitin, thereby mediating their sequestration into autophagosomes during Atg8-mediated autophagy [66]. In yeast, Hsp42 and Cue5 are required for the autophagic degradation of inactive proteasomes, with Hsp42 as a chaperone required for the sequestration of proteasomes into insoluble protein deposit (IPOD) sites, while Cue5 is responsible for linking ubiquitylated proteasomes to Atg8 [69]. Prolonged nitrogen starvation induces proteasome autophagy, with proteasomes shuttling from the nucleus to the cytoplasm. If the formation of autophagosomes is blocked during this process, CP components form cytoplasmic foci in a compartment referred to as agglomeration [65]. In yeast, sorting nexins Snx4/41/42 assist in the agglomeration of proteasomes en route to Atg-8-mediated autophagy [67]. However, the exact mechanism of proteasome accumulation during nitrogen starvation and the manner in which it is shuttled to the autophagosome remain unclear. However, this proteasome agglomeration may not constitute a proteasome condensate, given the proposed model of cytoplasmic puncta enclosed by an autophagosomal membrane [67]. Another interesting finding has shown the concurrent partitioning of proteasomes between autophagy and PSGs [37]. Further studies are needed to elucidate the mechanisms that determine whether the proteasome undergoes fate-specific breakdown or storage and to determine how previously identified cell signaling pathways, such as AMP-activated protein kinase (AMPK) and TORC1, regulate this process.

In senescence, researchers used live-cell imaging and tag-exchange approaches (e.g., Rpn11-Flag/EGFP knock-in mice) to demonstrate that older proteasomes (~3 days) appear to migrate to the cytoplasm for breakdown [58]. During amino acid starvation in mammalian cells, proteasomes translocate from the nucleus to the cytoplasm to enhance cytoplasmic proteolysis. Strikingly, supplementation with aromatic amino acids (tyrosine, tryptophan, phenylalanine, or YWF) can trigger apoptotic arrest in cancer cells, resulting in cell death. This process is mediated by the mTOR pathway and the addition of YWF. The supplementation of amino acids and mTOR activation inhibits proteasome translocation from the nucleus to the cytoplasm and arrests cytoplasmic proteasome activity and autophagy, leading to the buildup of cytotoxicity and initiating apoptosis [60]. Approximately 30% of newly synthesized proteins originate from previously degraded proteins by the proteasome [70]. Upon proteasome inhibition with MG132, the accumulation of ubiquitylated proteins increased significantly, thereby stunting amino acid recycling. During acute leucine and methionine deprivation, proteasome inhibition led to rapid depletion of amino acid concentrations and translational impairment [71]. These studies provide insights into the importance of proteasome translocation dynamics in maintaining cellular proteostasis under stress conditions. Carbon starvation results in the microautophagy and macroautophagy of proteasomes in yeast cells [72]. Carbon starvation can concurrently initiate the formation of PSGs while inactive or assembly-defective proteasomes are sorted for microautophagic breakdown. Carbon starvation-induced proteasome autophagy follows different mechanisms from nitrogen starvation, where AMPK and endosomal sorting complex required for transport (ESCRT) drive carbon starvation-induced microautophagy that recruits aberrant proteasomes into the vacuole [37,41]. Additionally, when microautophagy is inhibited by removing the AMPK subunits, the CP-containing proteasome condensates congregate into IPODs to restrict proteotoxicity in mother cells [37].

**Figure 1:**
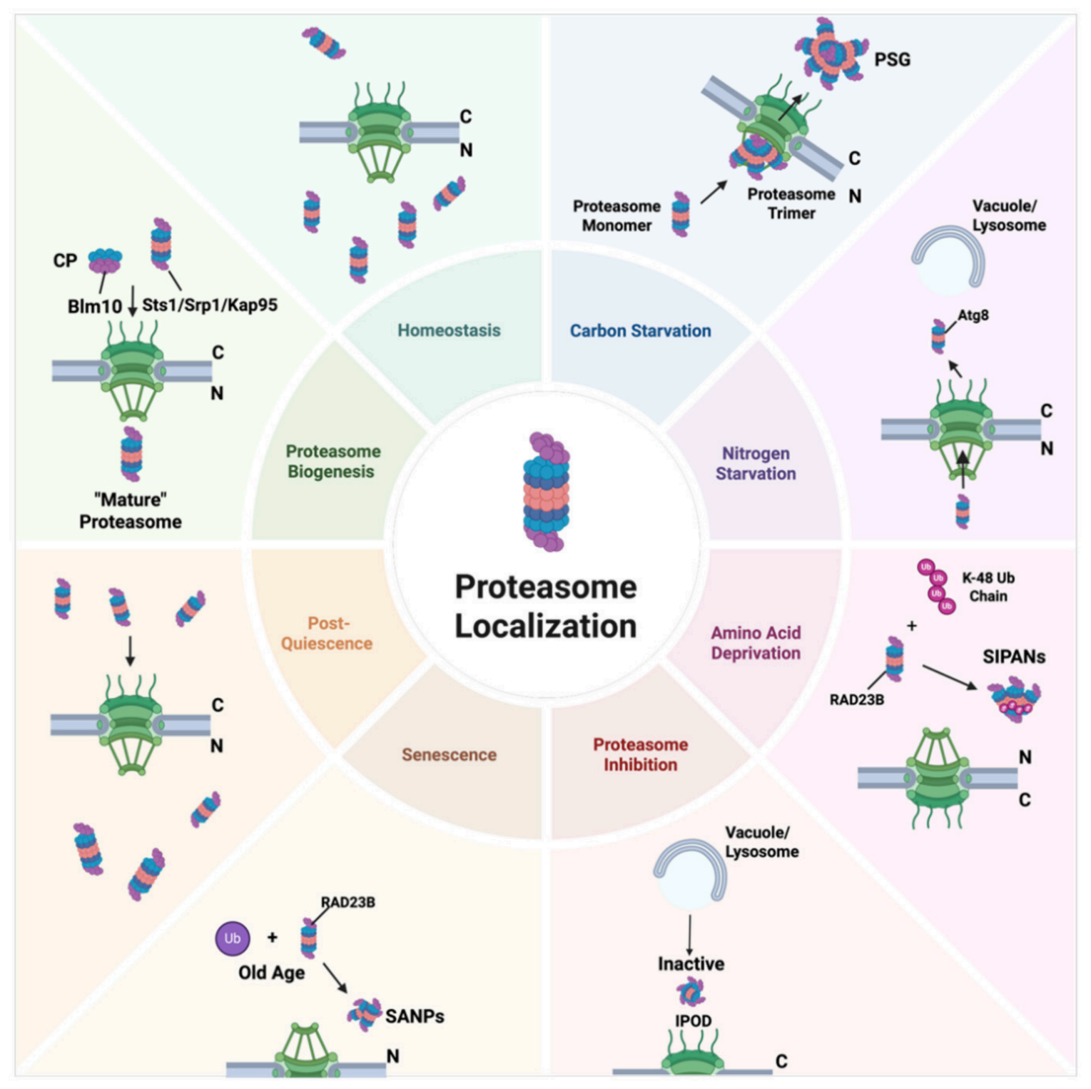
Overview of proteasome localization. Presented are representative events in proteasome translocation dynamics as described in the main text. The figure depicts instances of proteasomes sequestered in the nucleus (e.g., homeostasis, amino acid deprivation, and senescence); instances of proteasomes shifting from the cytoplasm to the nucleus (e.g., proteasome biogenesis and post-quiescence); instances of proteasomes shifting from the nucleus to the cytoplasm (e.g., carbon starvation); and instances of proteasomes translocating to the vacuole for degradation (e.g., nitrogen starvation and proteasome inhibition). This figure was created in BioRender. Butcher, C. (2026) https://biorender.com/b517mb7 (accessed on 20 June 2026).

Understanding proteasome localization dynamics can provide insight into the contingency plans of cellular proteostasis during times of stress. Proteasome localization dynamics can shift with metabolic state (Figure 1). While a proper distribution of nucleocytoplasmic proteasomes helps maintain proteostasis under stress, the localization pattern does not necessarily mean that proteasome catalytic activity will increase or decrease concurrently. It is important to note that proteasome translocation dynamics may provide useful feedback for developing therapeutic targets for diseases related to proteasome dysfunction. Additionally, gaps remain in the understanding of proteasome translocation dynamics, including how proteasomes are shuttled across the nuclear membrane and the cellular factors involved.

## 3. Cellular Regulation of Proteasome Condensate Formation and Dissolution

Proteasome condensates are dynamic and reversible subcellular compartments that contain proteasome species and associated factors, which can form via LLPS [73]. Proteasome condensates can reversibly assemble and disassemble in response to nutrient availability. Not every proteasome condensate is created equal in structure and location. Although the structure and components of proteasome condensates vary under different stress conditions, they usually associate with proteasomes, ubiquitin, ubiquitylated substrates, and/or shuttle factors (RAD23B, Dsk2/UBQLN2) [40,59,74,75,76]. Currently, the mechanistic basis for proteasomes congregating into condensates is unclear. However, recent progress in proteasome condensate research has shed light on potential hypotheses.

Proteasome condensate formation requires the assistance of multiple proteasome shuttle factors and ubiquitin chains [75]. In yeast, deletion of either Rad23 or Dsk2, both prominent proteasome shuttle factors, inhibited proteasome condensate formation, while double deletions blocked condensate formation under several tested stress conditions [75]. Moreover, Rad23 and Dsk2 also colocalized with proteasome condensates. Notably, Ddi1 deletion, a third shuttle factor, led to the formation of proteasome condensates during exponential cell growth [75]. However, Ddi1 was inconsistent in colocalization with proteasome condensates, and proteasome condensate formation still required the presence of Rad23 and Dsk2. K48 ubiquitin chains played an important role in the formation of proteasome condensates. K48 chains may act as a scaffold for proteasome shuttle factors, allowing for a network of proteins to form without relying on a molecular membrane [75]. Notably, the LLPS of UBQLN2, a Dsk2 homolog in mammalian cells, is regulated by polyubiquitylation chain types [77]. Interestingly, *in vitro* analysis of K63 and K48 ubiquitin chains in proteasome condensate formation revealed that both K63 and K48 linkages can induce UBQLN2-associated proteasome condensate formation, although the K48 linkage showed a weaker effect [78]. The binding partners are located on the proteasomes themselves, and the driving factors for the inclusion of K48 chains and Dsk2 in proteasome condensates remain unclear. Based on current research, the K48 chain may serve as a molecular tether to support congealment of proteasome components during phase transition and/or accruement of other proteasome components in the cytoplasm. Dsk2 may function as a padlock system during proteasome condensate accumulation, thereby regulating condensate size [78].

**Figure 2:**
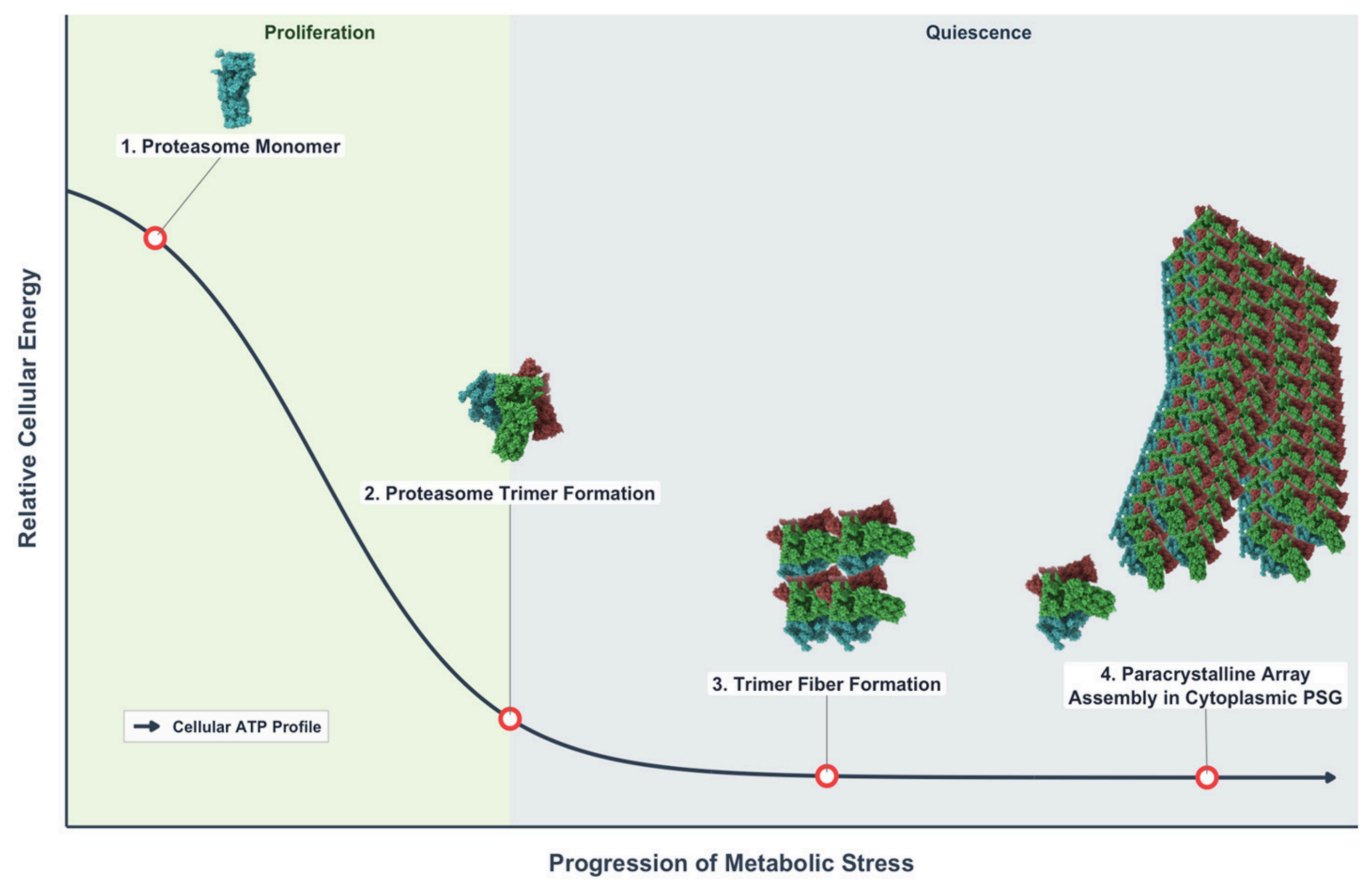
Overview of proteasome storage granule formation. Representative structures for proteasome storage granule (PSG) formation in yeast cells. Upon carbon starvation, proteasome monomers link together to form proteasome trimers. Proteasome trimers then align near the nuclear periphery and form proteasome fiber bundles. Proteasome fiber bundles then assemble into paracrystalline arrays in PSGs. ChimeraX 1.12 was used to visualize and prepare the proteasome trimer (PDB: 9IWR) [62,79]. Proteasome monomer is individually colored in the trimer.

How PSGs form in yeast and their mechanistic progression from the nucleus to the cytoplasm remain relatively new in the proteasome field. Recently, *in situ* cryo-electron tomography (cryo-ET) of PSGs has demonstrated a structural progression of proteasome condensates from a monomeric state to paracrystalline packing (Figure 2) [62]. Upon entering quiescence, doubly capped 30S proteasome monomers assemble into proteasome trimers and align adjacent to the NPC for nuclear export. Strikingly, the exposed surfaces along the alpha-helices from the three N-terminal tetratricopeptide repeats (TPR1-3) of Rpn9 contact parts of the surface of Rpn5, Rpn6, and Rpn7 on adjacent proteasomes in the trimer, indicating the importance of Rpn9’s TPR regions in trimer formation. This was confirmed via mutagenesis of the Rpn9 TPR region, which disrupted PSG formation. Proteasome trimers then bundle into a quaternary structure. The higher-order structure then forms paracrystalline arrays in proteasome condensates. The importance of NPC in proteasome translocation was confirmed using Mlp1/2, NPC basket proteins, with mutant strains showing decreased formation of proteasome condensates [62]. However, the specific binding partners of proteasome translocation remain undiscovered.

Additional factors are involved in PSG formation, such as cytosolic pH, ubiquitin, ESCRTs, and the SNARE protein Pep12 [37,39,74,80]. Cytosolic pH can dictate PSG formation, with drops in pH due to mutated V-ATPase, an important regulator of pH homeostasis, resulting in PSG formation without carbon starvation [39]. When a carbon source is resupplied to the cell, PSGs dissolve, and proteasomes reimport into the nucleus [81]. Moreover, AMPK, along with ESCRTs, directs low glucose-induced microautophagy of aberrant proteasomes, thereby promoting reversible PSG formation [37]. Meanwhile, the AMPK kinase functionally regulates the phosphorylation status of proteasomes. This post-translational modification is likely an indirect regulation of proteasome phosphorylation states [64]. While proper phosphorylation is required for the dissolution of proteasome condensates, the specific phosphorylation sites remain unclear. Interestingly, under long-term glucose starvation, removal of AMPK led to the accumulation of persistent proteasome condensates that were resistant to dissolution upon glucose refeeding [37,64]. While AMPK is required for the dissolution of PSG during glucose recovery, several questions remain unanswered. For instance, why are these persistent granules time-dependent? What other factors might contribute to the rigid structures preventing dissolution? Does mammalian AMPK play a similar role in proteasome condensate dissolution? Could AMPK functionality be used in the development of therapeutic targets for disease treatment? For PSGs, it remains unclear what the translocation requirements for proteasome condensates are during shifts in metabolic aptitude and what dictates where proteasomes should translocate under different stress conditions, since carbon stress directs proteasomes to the cytoplasm while senescence triggers proteasomes to remain or import into the nucleus. Additionally, whether the higher-order proteasome supramolecular structures, paracrystalline arrays, are generalized to yeast or applicable to human cells is also unknown.

Beyond yeast PSGs, mammalian cells deploy nuclear proteasome condensates depending on the specific stressor. Two well-characterized nuclear assemblies include SANP and SIPAN [40,59]. These two types of proteasome condensates highlight potential localization differences between mammalian and yeast proteasome stress responses.

Under cellular senescence, mammalian proteasomes assemble into SANPs to maintain nuclear proteostasis during growth arrest [59]. SANPs are liquid-like condensates that require active 26S proteasomes, K48 ubiquitin chains, and RAD23B to support their formation. In this context, RAD23B acts as an essential driver of phase separation, with RAD23B knockout completely abolishing SANP assembly [59]. Functionally, SANP formation prevents toxic mitochondrial dysregulation and restrains reactive oxygen species (ROS) buildup.

Similar to senescence, amino acid starvation induces the formation of proteasomes into SIPAN near the nuclear periphery [40]. SIPAN selectively enriches K48-linked polyubiquitin chains, fully assembled proteasomes, deubiquitinase, and RAD23B. Interestingly, proteasome inhibition with MG132 accelerated SIPAN formation, which was associated with decreased cellular amino acid levels. As with other biomolecular condensates, SIPAN can dissolve upon nutrient replenishment. Overall, the presence of SIPAN in the cell is a hallmark of subsequent cell death. SIPAN formation promotes the congruent fitness of the system over the individual and might be in place to decrease competition during nutrient starvation [40].

Proteasomes and autophagy play complementary roles in maintaining protein turnover in cells. Proteasome inhibition with MG132 can result in the accumulation of aberrant proteins and significant defects in cell proliferation and functionality that require the autophagy system to circumvent apoptosis [82]. In yeast cells, active proteasomes were found to associate with the juxtanuclear quality control compartment (JUNQ) under cytotoxic stress [76]. Inactive proteasomes can be compartmentalized into specialized holding aggregates in yeast, referred to as IPODs. IPODs are normally formed when traditional PQC pathways (UPS and autophagy) are overwhelmed by proteotoxic stress [76]. IPODs work to alleviate proteotoxic stress via the allocation of terminally tagged misfolded or insoluble proteins into aggregates [69]. The partitioning of toxic substrates will alleviate direct harmful interactions with other cellular proteins [83]. Inactive proteasomes are shuttled from the nucleus to the cytoplasm, where they are passed off to the IPOD in an Hsp42-dependent manner [84], resulting in autophagic degradation. Histone deacetylase 6 plays an important role in the regulation of aggresome formation, as it can bind to both polyubiquitylated misfolded proteins and dynein motors, thereby transporting the misfolded proteins for aggresome formation [85].

Hyperosmotic stress, or hypertonic stress, is induced when the extracellular solute concentration surrounding the cell is higher than that of the intracellular components, causing cellular shrinkage and potential cell death if not properly adapted [86]. In the HCT116 colon cancer cell line, proteasomes were shown to form dense nuclear foci, referred to as osmotic-induced proteasome condensates via LLPS. Currently, osmotic-induced proteasome condensates have been shown to form during disruption of nuclear-cytoplasmic transport and contain structurally altered proteasomes due to osmotic stress. Similar to other proteasome condensates, osmotic-induced proteasome condensates are reversible and restore their normal function when stress is relieved [73,87].

In *A. thaliana*, proteasomes were shown to sequester into cytosolic aggregates known as stress granules (SGs) upon heat stress. SGs were composed of proteasomes, RNA-binding proteins, and mRNA. SGs are thought to serve as a storage and protective agent against mRNA breakdown [88]. Proteasomes play a role in the disassembly of SGs when heat stress is relieved. Functional studies of proteasome inhibition confirmed that proteasome activity was required for the disassembly of SGs upon exiting heat stress conditions. Additionally, proteasome proteolytic activity increased when the proteasome was present in SGs. This functionality could be attributed to two factors: an increased total concentration of proteins in the SGs or an overexpression of a proteolytic subunit’s activity [89]. What remains unclear is the exact function of proteasomes during SG disassembly. Whether proteasomes actively degrade the components involved in the dissolution of SGs also remains unknown.

**Figure 3:**
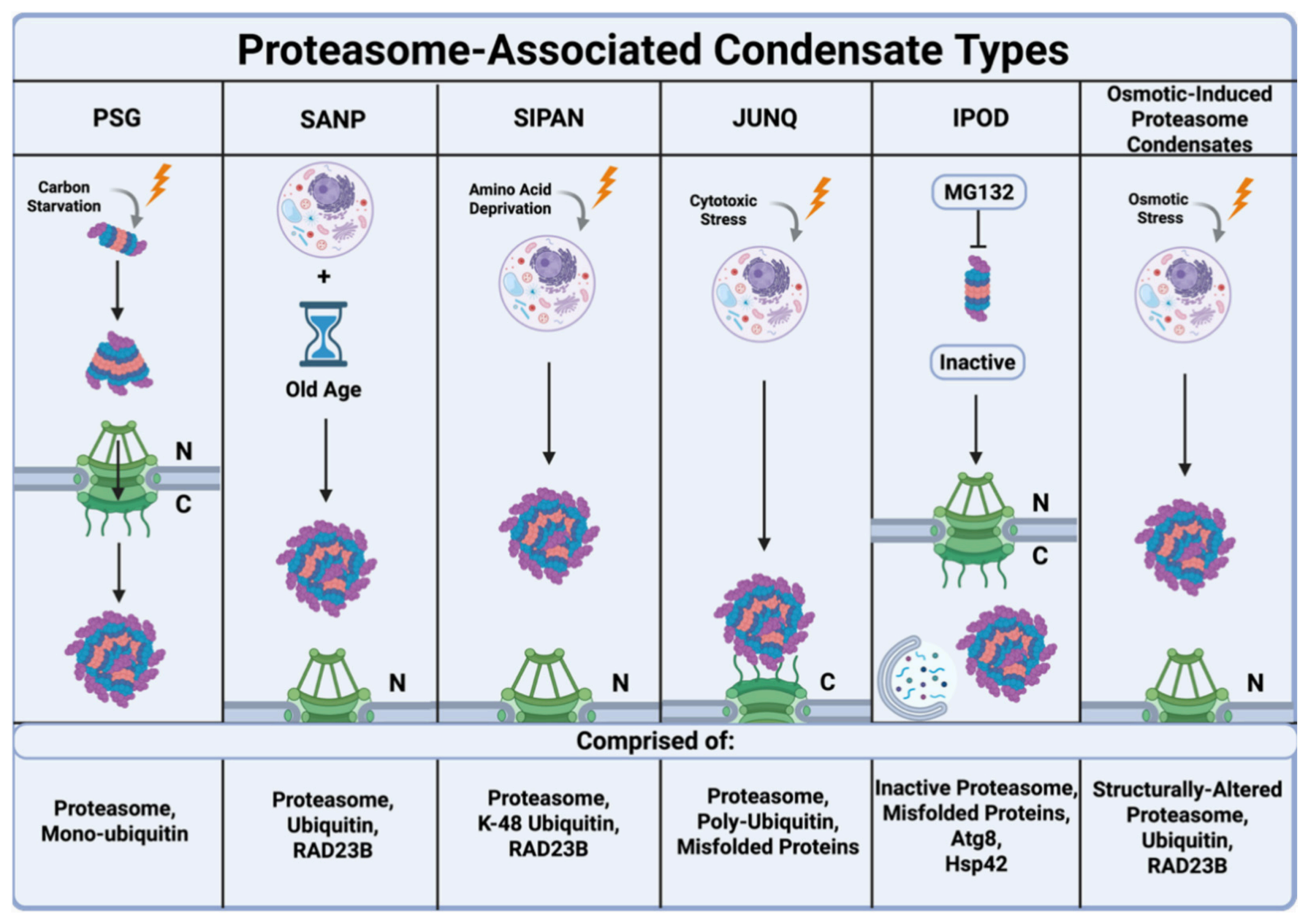
Proteasome condensate overview. Presented are different types of proteasome-containing condensates and their known components. See main text for further details. Post-stress signaling proteasomes are shown to form proteasome agglomerates. The type of stress can determine the location and components of the proteasome condensates. PSGs are induced via carbon starvation, resulting in a cytoplasmic proteasome condensate. SANPs are induced in older-age proteasomes, resulting in nuclear proteasome condensates. SIPANs are induced via amino acid deprivation, resulting in nuclear proteasome condensates. Cytotoxic stress can induce the association of active proteasomes with JUNQs for misfolded protein clearance. Proteasome inhibition can lead to the association of inactive proteasomes with IPODs. Osmotic stress can result in the formation of nuclear proteasome condensates. This figure was created in BioRender. Butcher, C. (2026) https://BioRender.com/2ud27o7 (accessed on 20 June 2026).

Overall, the structure and composition of proteasome condensates can vary drastically depending on multiple factors (Figure 3). Currently, there is limited knowledge of why proteasome condensates form and of their biological roles in the cellular response to shifts in metabolic state. Furthermore, how proteasome condensates interact with other biomolecular condensates during stress conditions remains unclear. For example, previous studies on stress granules in *A. thaliana* have demonstrated that proteasome caspase-like activity regulates SG assembly [90]. In contrast, cryo-ET structures indicated that proteasomes were inactive in PSGs in yeast [62]. If both exist, how are these two events related, and what associated factors or mechanisms might dictate their action?

## 4. Clinical Significance

Proteasomes play a crucial role in the removal and recycling of proteins in the cell. Proteasome dysfunction is a hallmark of many different human diseases, including neurodegenerative disorders and proteasome-associated autoinflammatory syndromes (PRAAS) [91,92,93]. While proteasome function is required to prevent certain diseases, the proteasome can be exploited by cancer cells as a regulator of carcinogenesis [94]. Multiple myeloma (MM), a plasma cell cancer, is an incurable disease that leads to low blood cell counts, bone lesions, and hypercalcemia. The lifespan of those diagnosed with MM can vary based on age, genetics, treatment response, and severity of the cancer [95]. A prevalent treatment option for extending the life expectancy of MM patients is proteasome inhibitors. In MM, proteasome inhibitors disrupt several common cellular pathways, including NF-κB signaling, protein degradation, and angiogenesis, leading to the accumulation of ubiquitylated proteins and apoptosis. Experimentally, the inhibition of proteasome activity can be clinically monitored by the rapid accumulation of K48-linked polyubiquitin chains [96]. Prior to the clinical introduction of proteasome inhibitors, the median survival of MM patients was about 3 years. Modern therapeutic treatments, including proteasome inhibitors, have significantly increased the median life expectancy of MM patients [97]. There are currently three FDA-approved proteasome inhibitors for clinical use, including bortezomib (Velcade), introduced in 2003; carfilzomib (Kyprolis) in 2012; and ixazomib (Ninlaro) in 2015 [98]. Unfortunately, MM patients can develop drug resistance. To combat this, researchers found that the coupled inhibition of Crm1 (XPO1), a nuclear export component, via its clinical inhibitor, selinexor, was able to bypass drug resistance to bortezomib or carfilzomib [99]. This finding drives a connection between a translocation-based biomarker and clinical drug discovery. The current clinical understanding of proteasomes is that they are required for conserved and diverse cellular functions but also can be targeted therapeutically for certain diseases.

Chronic atypical neutrophilic dermatosis with lipodystrophy and elevated temperature (CANDLE) syndrome is an autoinflammatory disease typically diagnosed during infancy. Patients with CANDLE syndrome will typically suffer from recurrent fevers, rashes, lesions, and contractures. CANDLE syndrome results from a mutation in the human immunoproteasome CP subunit PSMB8, a catalytic subunit responsible for processing antigens for MHC class I antigen presentation [100]. Interestingly, the yeast ortholog Pre2 regulates standard chymotrypsin-like activity [101]. In 2012, a clinical trial was conducted using patients diagnosed with CANDLE syndrome. Researchers found that in each patient, PSMB8 was mutated, albeit in different locations. In addition to PSMB8 mutations, they also found each patient expressed high levels of interferon-γ-inducible protein 10, indicative of an active immune system [102,103]. How proteasome translocation dynamics and the impact of proteasome condensate formation relate to the pathogenesis of modern diseases, or their role in potential therapeutics, remain largely unexplored.

We propose that targeted disruption or activation of proteasome functionality could be programmed as a potential therapeutic strategy for treating diseases. For example, in Alzheimer’s patients, proteasomes were shown to localize to the sites of most dystrophic neurites, with proteasome downregulation initiating proteostatic failure and the accumulation of amyloid-beta and hyperphosphorylated tau. Targeting proteasome localization and upregulation in those locations may ease the progression of Alzheimer’s; however, future studies are needed to validate this therapeutic strategy. Understanding the causes of decreased proteasome function could provide insight into potential therapeutic targets or a biomarker for assessing Alzheimer’s patients [104,105].

A major limitation of current research is the extent to which the cellular regulation of proteasome localization and condensate formation is conserved across human cell types. It remains less evident that proteasome condensates serve to protect the proteasome from autophagic degradation during metabolic fluctuations. Based on current evidence, proteasome condensates serve not only as proteolytic regulators that facilitate rapid cellular recovery but also as regulators of proteasome function upon alleviation of metabolic stress. Also, the clinical implications of targeted spatiotemporal control of proteasome components and proteasome-associated factors merit further investigation to remodel the cellular proteostasis network. Previous studies have shown that the inhibition of proteasome translocation by Sestrin3, a mediator of YWF sensing and essential for proteasome translocation in HeLa cells, resulted in tumor suppression [60]. Moreover, it remains unclear whether the survival-promoting effects of inhibiting or promoting stress-induced proteasome nuclear export vary across different cell types. How the inhibition or activation of proteasome function adversely affects other nearby cells remains to be determined.

## 5. Conclusions

The proteasome is an evolutionarily conserved protease complex that can shuttle across cellular compartments in response to metabolic need. Proteasome translocation exhibits variability that is influenced by multiple factors, including species, cellular age, intracellular pH, and environmental conditions. The present knowledge of proteasome nucleocytoplasmic translocation is expanding, with the nuclear import pathway better studied than the export mechanisms. The proteasome nuclear import pathway can utilize the Sts1/Srp1/Kap95 canonical importin α/β pathway in yeast or the AKIRIN2 pathway in mammalian cells during proteasome biogenesis, while Blm10 plays an important role in proteasome nuclear reimport during PSG dissolution. Proteasome nuclear export is currently limited to the canonical export receptor Crm1/Xpo1 and the UBIN-POST system. Despite the advanced cryo-ET structures of the PSG formation process, information about additional export pathways and contributors remains poorly understood. There are also stress-specific pathways for proteasome translocation, each of which may involve distinct pathways to efficiently shuttle proteasome species. Environmental and physiological stressors, such as carbon starvation, amino acid deficiency, hyperosmotic stress, and cellular senescence, serve as primary metabolic signals to initiate proteasome redistribution among different subcellular compartments. Under certain stress conditions, proteasomes reorganize into proteasome condensates via LLPS. However, proteasome condensate localization and phase transition mechanisms vary based on the stressor. One of the well-characterized conditions is carbon starvation, where, upon the switch to quiescence, inactive 30S proteasome monomers can form trimer structures. The proteasome trimers can assemble into fibers/bundles that are further packed into paracrystalline arrays in PSGs (Figure 2). PSGs are likely used as a rapid proteasome control center that allows the proteasome to conserve energy under stress conditions and redistribute to sites of need upon exit from quiescence. In contrast, both amino acid-deprivation-induced SIPANs and senescence-induced SANPSs are found in the nucleus.

Proteasome translocation dynamics and proteasome condensate formation pathways are relatively underexplored avenues for regulating proteasome function. The field is also limited by the shortcomings of primary research systems. While yeast models excel at demonstrating autophagic coupling and PSG dynamics, they lack intercellular signaling networks at the tissue and organismal levels found in higher eukaryotes. Conversely, mammalian and plant models are constrained by technical difficulties in examining proteasome translocation dynamics *in vivo*. Resolving these species-specific variations, for instance, why proteasome condensates preferentially assemble in the cytoplasm in yeast models but favor the nucleus in mammalian cell models, will be critical for a better understanding of proteasome translocation dynamics. Although finding a model organism in nature that combines the advantages of yeast and mammalian systems may be challenging, it is possible to create a versatile research system with the advancements of synthetic biology and systems biology to meet the needs in future proteasome studies. Bridging these functional gaps in proteasome translocation dynamics will usher forth the development of next-generation proteasome-targeting therapeutics in the treatment of proteasome-related diseases.

## Data Availability

No new data were created or analyzed in this study. Data sharing is not applicable to this article.
